# Synthesis and Characterization of an Antioxidative Galactomannan–Iron(III) Complex from *Sesbania* Seed

**DOI:** 10.3390/polym11010028

**Published:** 2018-12-25

**Authors:** Caoxing Huang, Yuheng Tao, Min Li, Weiyu Zhang, Yimin Fan, Qiang Yong

**Affiliations:** Co-Innovation Center for Efficient Processing and Utilization of Forest Products, College of Chemical Engineering, Nanjing Forestry University, Nanjing 210037, China; hcx@njfu.edu.cn (C.H.); tyh0305@njfu.edu.cn (Y.T.); szxapy@163.com (M.L.); wyzhang180912@163.com (W.Z.); fanyimin@njfu.edu.cn (Y.F.)

**Keywords:** galactomannan, *Sesbania* seed, galactomannan–iron(III) complexes, antioxidant, bioavailability

## Abstract

Galactomannan, a water-soluble polymer in the cell wall of leguminous plants, has been proven to possess anticancer and antioxidative activity. In this work, galactomannan with different molecular weights (GM-40 and GM-65) was obtained from *Sesbania* seeds and synthesized into galactomannan–iron(III) complexes, which are termed as GM-40-Fe and GM-65-Fe, respectively. These galactomannan–iron(III) complexes are intended to function as organic iron supplements to treat iron deficiency with the added benefit of antioxidative activity. The prepared galactomannan–iron(III) complexes were characterized for chemical composition, morphology, antioxidant capacity, and bioavailability in vitro. The results showed that galactomannan–iron(III) complexes could be produced with iron contents as high as 65.4 mg/g. Antioxidant assays indicated that both GM-40-Fe and GM-65-Fe exhibited antioxidant activities for scavenging radicals in vitro. The iron release/bioavailability assays showed that the iron was easily released into artificial gastric and intestinal juices, resulting in iron release rates of 88–94% over 300 min. These results suggest that galactomannan–iron(III) complexes synthesized from *Sesbania* seed polysaccharides are capable of being administered as organic iron supplements to patients with iron deficiency.

## 1. Introduction

The mineral iron plays a fundamental role in maintaining human health, and is involved in the metabolism, immune functions, and many other biological systems. Chronic iron deficiency, diagnosable as iron deficiency anemia (IDA), is a globally-pandemic nutritional disease that mostly affects children (~5–14 years old) and pregnant women [1,2]. It is speculated that inadequate iron intake from the daily diet coupled with excessive loss of iron are the leading causes of IDA diagnoses. Oral administration of iron supplements is the most-often prescribed means to treat and prevent IDA [3].

There are two distinctive categories of iron supplements: inorganic iron and organic iron. Inorganic irons, such as ferrous sulfate, ferrous fumarate, and ferrous gluconate, can treat IDA by ingestion [4]. However, it has been reported that some patients may suffer symptoms of epigastric pain, diarrhea, and constipation as side effects if they take too much inorganic iron [5]. In contrast, organic iron is regarded as the superior iron supplement due to recent advances in the development of supplements. Organic iron’s superiority is attributable to its higher bioavailability, lower propensity to accumulate and cause iron overdose, better stability in the supplement prior to ingestion, and incitation of few sides effects following ingestion [6,7].

Chemically, organic iron supplements are atomic iron complexed with proteins, polysaccharides, or other organic ligands. Polysaccharide–iron(III) complexes are the most widely-studied type of organic iron supplement [8,9]. In polysaccharide–iron(III) supplements, the polysaccharide element of the complexes prevent the iron from being rapidly hydrolyzed and solubilized. This results in a more controlled system of iron delivery to the human body. As an additional benefit, the polysaccharides tend to be generally derived from edible terrestrial or aquatic biomasses, resulting in the formulated polysaccharide–iron(III) complexes being a much safer means for treating IDA (compared to inorganic iron supplements). Iron–dextran and iron–sucrose complexes have been clinically used to treat patients with iron deficiency for over 30 years [10,11], however new types of organic iron complexes using polysaccharides from functional plants are still under research and development.

According to a recent report, it was found that polysaccharides from *Astragalus membranaceus* [5], *Enteromorpha prolifera* [12], and the mushroom *Inonotus obliquus* [2] are potential candidates for preparing less biologically-hazardous polysaccharide–iron(III) complexes. These newly investigated complexes have demonstrated additional beneficial bioactivities beyond those of iron, including anti-tumor and anti-oxidation activity. It was speculated that these additional benefits are derived from the original biological activities of the component polysaccharides. Regarding polysaccharide–iron(III) complexes, the polysaccharide acts as the ligand and non-specifically interacts with the Fe^3+^ (electron acceptor) to form polysaccharide–iron(III) complexes that can be administered as organic iron supplements [11,12]. Recently, it was found that the galactomannan from *Sesbania* seed has been identified as a non-noxious and bioactive polysaccharide for living organisms [13,14]. These findings could prove beneficial if such polysaccharides were utilized in organic iron supplement formulations. However, there are no reports to date that seek to be gauge whether these beneficial properties of galactomannan from *Sesbania* seed are carried through when used as complexing agents in organic iron supplements. Hence, the valorization the galactomannan from *Sesbania* seed as an iron supplement could open a new, innovative pathway to providing the supplement to treat and prevent IDA. 

**Figure 1:**
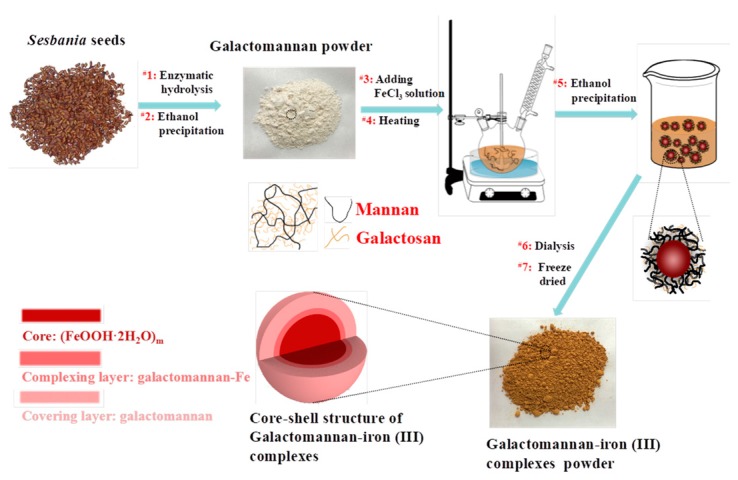
Flow chart of the synthesis the galactomannan–iron(III) complexes from *Sesbania* seed.

In this work, galactomannan–iron(III) complexes were prepared from galactomannans obtained from *Sesbania* seeds using the process that is visually described in Figure 1. Galactomannans of different molecular weights were compared as complexing agents. Once produced, the galactomannan–iron(III) complexes were synthesized and characterized by their chemical components, physicochemical features, and morphology. In addition, the antioxidant activities and bioavailability of the prepared galactomannan–iron(III) complexes were evaluated by radical-scavenging (DPPH and hydroxyl radicals) and iron-releasing assays in vitro. The overall goal of this work was to demonstrate that the galactomannan from *Sesbania* seeds can be beneficially applied as a component of organic iron supplements with antioxidative activity.

## 2. Materials and Methods

### 2.1. Materials

The *Sesbania* seeds were provided by Jiangsu Kangwei Biologic Co., Ltd. in Yancheng, China. The *Sesbania* seeds were ground into a particle size (20–80 mesh) prior to all experiments. The chemical composition of the *Sesbania* seeds was quantified according to the laboratory analytical procedure from the National Renewable Energy Laboratory [15] and results were 95.1% of galactomannan, 3.0% of protein, and 1.7% of ash.

### 2.2. Preparation of Galactomannan–Iron(III) Complexes

Galactomannan (GM) powder was isolated from the *Sesbania* seeds according to our previous work [13]. Specifically, 100 g of *Sesbania* seed particles were mixed with 3 L 95% (*v*/*v*) ethanol and heated at 70 °C for 15 min to solubilize the proteins. Next, the seed particles were collected and mixed with 500 mL acetate buffer (50 mM, pH 4.8) for enzymatic hydrolysis. The enzymatic hydrolysis assay was conducted in a shaking incubator at 50 °C and 150 rpm for 24 h using β-mannanase for bioconversion (20 U/g GM) [13,16]. The hydrolysate was collected and its solutes were precipitated through the gradual addition of ethanol. The final concentrations of ethanol were 40% and 65% (*v*/*v*). The precipitated galactomannans were washed by the corresponding concentration of ethanol (40% or 65%) 3 times and freeze-dried to prepare galactomannan powder, termed GM-40 and GM-65, with respect to the final ethanol concentrations.

The galactomannan–iron(III) complexes were prepared according to the method of Shi et al. [7] with a minor modification. Specifically, 2 g of galactomannan powder (GM-40 and GM-65) was mixed with a certain amount of sodium citrate (galactomannan/sodium citrate ratio 2:1~6:1) and dissolved into 60 mL of deionized water. The solution was neutralized and next heated to the target temperature (50–90 °C). After reaching the specified temperature, 2 M FeCl_3_ and 20% NaOH (which raises the pH to 8.0–8.5) were slowly and concurrently added into the galactomannan solution until a brown powder appeared. The suspension was then heated at the specified temperature for 2 h. After the reaction, the solution was centrifuged at 5000 rpm for 10 min to separate the brown powder from the suspension, and next dialyzed by a dialysis membrane (molecular weight cutoff of 3000 g/mol) to remove unbound ions. The dialyzate was then mixed with 180 mL of 95% ethanol to precipitate the galactomannan–iron(III) complexes. The precipitate was recovered through centrifugation and then freeze-dried to obtain the powdered galactomannan–iron(III) complexes. The galactomannan–iron(III) complexes obtained from the GM-40 and GM-65 at optimum conditions are referred to as GM-40-Fe and GM-65-Fe.

### 2.3. Quantification of the Iron and Polysaccharides Content of the Galactomannan–Iron(III) Complexes

The galactomannan contents in GM-40, GM-65, GM-40-Fe, and GM-65-Fe were measured according to the procedure developed by the National Renewable Energy Laboratory (NREL) [15]. Specifically, 0.3 g of powder was subjected to 72% H_2_SO_4_ for hydrolysis for 1 h, following by diluting to 4% H_2_SO_4_ and autoclaving at 121 °C for 1 h. The monosaccharide concentrations in the hydrolysate were quantified using the high performance anion exchange chromatography (HPAEC) system equipped with a PA10 column (2 × 250 mm) and a pulsed amperometric detector. Deionized water and 3 mM NaOH were used as the eluent at a flow rate of 0.2 mL/min using the gradient elution program according to our previous work [17].

The iron contents in the prepared galactomannan–iron(III) complexes were quantified using the phenanthroline method, which involves UV-Vis spectroscopy [18,19]. Specifically, 10 mg of complex powder was mixed with 2 mL of 2 M HCl in volumetric flasks (50 mL) and then sonicated for 1 h to release the complexed iron. After ultrasonic treatment, 1 mL of hydroxylamine hydrochloride (10%, *w*/*v*), 2 mL of phenanthroline (0.1%, *w*/*v*), 5 mL of 6 M NaOH, and 5 mL of sodium acetate trihydrate buffer solution (pH 5.0) were successively added into the volumetric flask. Finally, deionized water was added to the flask to reach 50 mL of solution. The concentration of iron in the solution was determined by UV-Vis spectrometer at 510 nm and calculated according to the calibration curve derived from the standard solutions of ferrous ammonium sulfate. The iron content in the galactomannan–iron(III) complexes was then back-calculated based on the iron concentrations quantified.

### 2.4. Molecular Weight Analysis of the Galactomannan–Iron(III) Complexes 

The molecular weights of GM-40, GM-65, GM-40-Fe, and GM-65-Fe were estimated using a gel permeation chromatography (GPC) instrument equipped with tandem columns of Waters Ultrahydrogel TM 2000 (7.8 × 300 mm), Waters Ultrahydrogel TM 250 (7.8 × 300 mm) and Waters Ultrahydrogel TM 120 (7.8 × 300 mm) columns, which is accordance with the experiment in our previous work [13]. Deionized water was used as the eluent at the flow rate and temperature of 0.6 mL/min and 65 °C, respectively. Dextran T-series (1000–670,000 g/mol) were used as calibration standards to provide estimates of each sample’s molecular weight.

### 2.5. Characterization of the Galactomannan–Iron(III) Complexes

The morphological features of the galactomannan–iron(III) complexes were analyzed by TEM using a JEM 2100 (JEOL Ltd., Tokyo, Japan) and SEM-Mapping using FEG Quanta 400 (HITACHI Ltd., Tokyo, Japan) at accelerating voltages of 200 kV and 15 kV. 

The vibrations of molecules and polar bonds in the galactomannan–iron(III) complexes were investigated by Fourier transform infrared (FT-IR). FT-IR was performed using a Thermo Scientific Nicolet iN10 (Thermo Nicolet Corporation, Madison, WI) fitted with a liquid nitrogen-cooled MCT detector. The precipitates were analyzed in powder form and spectra were recorded using a spectral width of 400 to 4000 cm^−1^ at 4 cm^−1^ resolution and by accumulation of 128 scans. The thermogravimetric analysis of the galactomannan–iron(III) complexes were performed using a TGA thermal instrument (Perkin-Elmer, MA, USA). Approximately 10 mg of sample was heated from 30 to 600 °C at a rate of 10 °C/min in a nitrogen atmosphere with flow of 30 mL/min.

### 2.6. In Vitro Anti-Radiation Activity of the Galactomannan–Iron(III) Complexes

The antioxidant activities of galactomannan and the galactomannan–iron(III) complexes were quantified using assays by scavenging the 2,2-diphenyl-1-picryl-hydrazyl (DPPH) radical and hydroxyl radical in water solution. Assays were carried out in accordance with our previous work [17]. For the DPPH radical scavenging assay, 2.0 mL of DPPH (0.2 mM) ethanol solutions and 2.0 mL of anhydrous ethanol were mixed with 2 mL of galactomannan solution or galactomannan–iron(III) complex solution with different concentrations, respectively. After 0.5 h, the UV-absorbance of the mixtures was measured at 517 nm, with the measured value being termed as A_sample_ and A_control_. A total of 2.0 mL of anhydrous ethanol was mixed with 2 mL of DPPH (0.2 mM) ethanol solutions and absorbance of the mixtures was measured at 517 nm, termed as A_blank_. The DPPH radical scavenging ability (P) was calculated by following equation.

(1) $$P(\%)=\frac{1-(A_{\mathrm{sample}}-A_{\mathrm{control}})}{A_{\mathrm{blank}}}\times 100\%$$

For the hydroxyl radical scavenging assay, 1.0 mL of various concentrations of galactomannan solution or galactomannan–iron(III) complex solutions was first mixed with 2 mL of FeSO_4_ solution (9.0 mM) and 2.0 mL of salicylic acid-ethanol solution (9.0 mM). Next, 2.0 mL H_2_O_2_ (8.8 mM) was added into the above solution and incubated at 37 °C for 0.5 h. The absorbance of each reacted mixture was then measured at 510 nm, termed as B_sample_. Meanwhile, 1.0 mL of galactomannan solution or galactomannan–iron(III) complex solution with different concentrations were added into 2 mL of FeSO_4_ solution (9.0 mM), 2.0 mL of salicylic acid-ethanol solution (9.0 mM) and 2.0 mL of water. This new mixture was then allowed to react at 37 °C for 0.5 h. The absorbance of reacted mixture was then measured at 510 nm, termed as B_control_. Finally, 2.0 mL of water was mixed with 2 mL of FeSO_4_ solution (9.0 mM), 2.0 mL of salicylic acid-ethanol solution (9.0 mM) and 2.0 mL H_2_O_2_ (8.8 mM). The absorbance of the reacted mixture was measured at 510 nm, termed as B_blank_. The hydroxyl radical scavenging ability (R) was calculated using following equation.

(2) $$R(\%)=\frac{1-(B_{\mathrm{sample}}-B_{\mathrm{Bcontrol}})}{A_{\mathrm{blank}}}\times 100\%$$

### 2.7. Measuring the Iron Release Capability of the Galactomannan–Iron(III) Complexes

The bioavailability of the galactomannan–iron(III) complexes was evaluated by its iron-releasing ability in the stomach and intestines, simulated in artificial gastric juice (pH 2.0) and artificial intestinal juice (pH 8.0), which were prepared according to the methods described in Hasan et al. [20] and Dong et al. [21], respectively. To prepare the artificial gastric juice, 200 mg of sodium chloride and 320 mg of pepsin were dissolved into solution of 2 mL deionized water and 8 mL 1 M HCl. After dissolution, deionized water was added to reach the final volume of 100 mL. The final pH of this solution was measured to be ~2.0. To prepare the artificial intestinal juice, 900 mg of bile, 900 mg of pancreatin, 30 mg of trypsin, 40 mg of urea, 100 mg of NaHCO_3_, and 30 mg of KCl were dissolved into 100 mL deionized water. The final pH of this solution was measured to be ~2.0. Simulated digestion assays within the prepared artificial solutions were performed separately. During the release assays, 0.1 g of galactomannan–iron(III) complex was placed in 500 mL of gastric juice or intestinal juice and held in a flask. The flask was maintained at 37 °C and rotated at 100 rpm for 300 min. A total of 5 mL of the solution was then withdrawn at predetermined times, and an equivalent volume of the fresh artificial gastric juice/intestinal juice was added into the flask in order to maintain constant volume. The amount of released iron in the withdrawn aliquots was calculated based on the concentration of iron in solution, which was analyzed according to the phenanthroline method previously described in Section 2.3.

### 2.8. Statistical Analysis

All the measurements were conducted in triplicate and expressed as means ± standard deviation (SD) (SPSS 13.0).

## 3. Results and Discussion

### 3.1. Preparing of Different Molecule Weight Galactomannan from Sesbania Seeds 

In our previous work [13], we found that the seeds from *Sesbania* contain 22.1% of galactomannan, and this polysaccharide was demonstrated to show anticancer and other beneficial physiological activity. In this work, to further explore these properties of the polysaccharide–iron(III) complexes, two galactomannan fractionations with different molecular weights were obtained from the native galactomannan (isolated from *Sesbania* seed) by enzymatic hydrolysis with β-mannanase and gradual ethanol precipitation (ethanol at final concentrations of 40% and 65% (*v*/*v*)). As can be seen in Table 1, the galactomannans obtained from 40% ethanol precipitation (GM-40) and from 65% ethanol precipitation (GM-65) possessed different molecular weights. GM-40 was found to have a weight-average molecular weight (MW) of 14,700 g/mol, where GM-65 was 4890 g/mol, respectively. In addition, both GM-40 and GM-65 were mostly pure galactomannan preparations, with a galactomannan contents of 90.9% and 96.5%, respectively.

### 3.2. Determination of Key Factors Contributing to the Iron Content of Synthesized Galactomannan–Iron(III) Complexes

**Figure 2:**
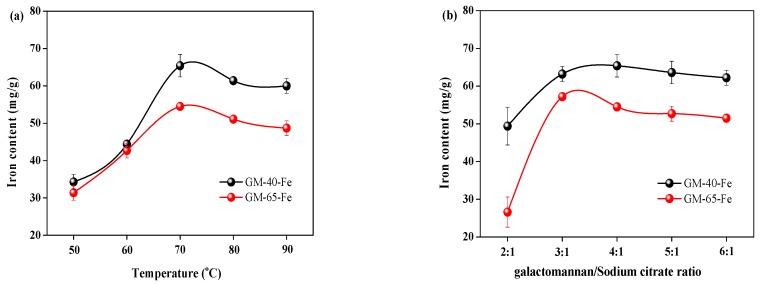
Complex synthesis around the variable temperature (**a**) and galactomannan/sodium citrate ratio (**b**) with respect to iron contents.

It is speculated that polysaccharide–iron(III) complexes consist of an iron–oxyhydroxide/oxide core (FeOOH) surrounded by the polysaccharide ligand. During the formation of polysaccharide–iron(III) complexes, both hydroxyl groups and carboxyl groups in the polysaccharide act as nucleation sites for the iron ions to form hydroxide bridges, which maintain the complexation [4]. Therefore, to produce a stable polysaccharide–iron(III) complex, both the polysaccharide and sodium citrate must serve as obligatory ligands to render complexed ferrous. The sodium citrate plays an indispensable role in initiating formation of the bridge between the polysaccharide and iron(III). It is also reported that the synthesis temperature is the dynamic factor influencing the properties of the polysaccharide–iron(III) complex [22]. To synthesize the polysaccharide–iron(III) complex, it is known that the pH should be 8–9, therefore additional investigation into the effects of pH was not carried out. Based on the aforementioned critical factors, both the reaction temperature and galactomannan/sodium citrate ratio were investigated to determine how they influence the iron content of prepared polysaccharide–iron(III) complexes. The results from these investigations are shown in Figure 2.

It can be observed in Figure 2a that increasing the reaction temperature from 50 °C to 70 °C led to an increase in the iron contents in the galactomannan–iron(III) complex produced with both GM-40 and GM-65 as the starting materials. Specifically, the iron contents increased from 34.3 mg/g and 31.4 mg/g to 65.4 mg/g and 54.5 mg/g for GM-40 and GM-65, respectively. When increasing the temperature from 70 °C to 90 °C, the iron contents in the galactomannan–iron(III) complex decreased in a similar fashion. This temperature-based phenomenon was also reported in the work of Chi et al. [12], who prepared similar complexes using polysaccharides from a different biological source. In the reported work, the iron content of the polysaccharide–iron(III) complexes obtained from *Enteromorpha prolifera* decreased when the synthesis temperature increased above 50 °C. This phenomenon may be due to higher temperatures causing changes to the spatial structure of galactomannan, which inhibited proper complexation from taking place. Another possibility may be that the elevated temperatures incited excessive molecular movement between galactomannan and iron(III), resulting less polysaccharide–iron(III) complexes being formed [23]. Therefore, it was concluded that a synthesis temperature of 70 °C was the optimum condition for using GM-40 and GM-65 to prepare galactomannan–iron(III) complexes.

Figure 2b displays the impact of sodium citrate on the iron content in the galactomannan–iron(III) complexes produced from GM-40 and GM-65. It can be seen that the iron contents in the galactomannan–iron(III) complexes increased with the increasing weight ratio of sodium galactomannan/sodium citrate (up to 4:1 for GM-40 and 3:1 for GM-65). Interestingly, beyond these points, a decreased iron content was observed. During the synthesis process of the polysaccharide–iron(III) complexes, sodium citrate serves as the bridge that fosters association between the polysaccharide and iron(III) [2,5]. This is a critical step for the synthesis of polysaccharide–iron(III) complexes, therefore sodium citrate is essential in the reaction system under investigation. However, an excessive dose of sodium citrate will likely reduce the pH of the synthesis medium below the target range of ~8.0–9.0. A lowered pH can cause the hydrolysis of complexed irons, with the net effect being finished complexes with decreased iron contents. Hence, the optimum ratios of galactomannan/sodium citrate for GM-40 and GM-65 were identified to be 4:1 and 3:1, respectively. At these ratios, the resultant galactomannan–iron(III) complexes contained iron contents of 65.4 mg/g and 57.2 mg/g, respectively. The galactomannan–iron(III) complexes produced from the GM-40 and GM-65 at the identified optimal conditions were termed GM-40-Fe and GM-65-Fe, respectively.

### 3.3. Characterization of the Galactomannan–Iron(III) Complexes

To characterize the synthesized galactomannan–iron(III) complexes, the molecular weights of GM-40-Fe and GM-65-Fe were estimated and the results are shown in Table 1. It can be seen that the molecular weights of GM-40-Fe and GM-65-Fe were 16,100 g/mol and 5320 g/mol, which were lower than that of the GM-40 and GM-65. This result demonstrates that the complexation of iron(III) with galactomannan resulted in a molecule that has a slightly increased molecular mass relative to the original galactomannan. These findings are consistent with the work of Chi et al. [12]. Another interesting observation involved the distribution of molecules, which was more homogenous for the complexes compared to the original polysaccharides. Specifically, the polydispersity index (PDI) of GM-40 and GM-65 decreased from 1.23 and 1.12 to 1.02 and 1.01, respectively.

**Figure 3:**
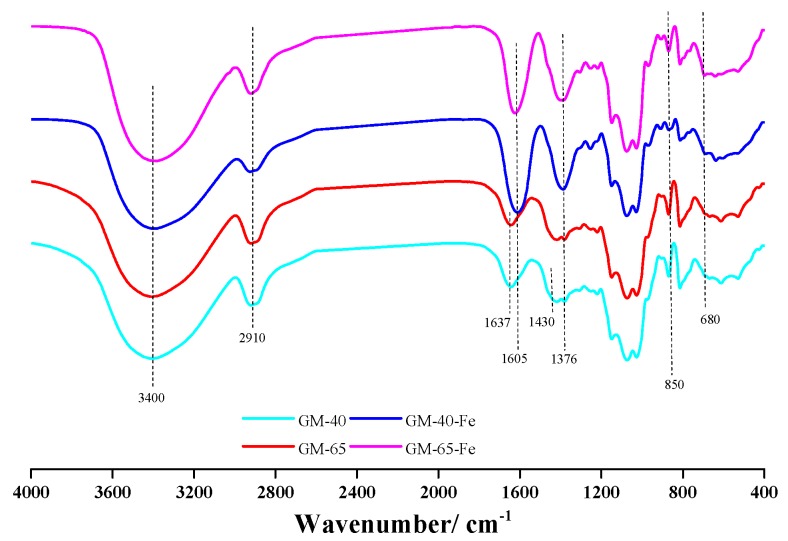
The FT-IR spectra of galactomannan and galactomannan–iron(III) complexes.

FT-IR analysis of the galactomannan–iron(III) complexes enabled the identification of the chemical structures in both the original polysaccharide structures, as well as the structures and bonds that were modified following the conversion of galactomannan to galactomannan–iron(III) complexes [2]. The FT-IR spectra of GM-40, GM-65, GM-40-Fe and GM-65-Fe are shown in Figure 3.

It can be observed that GM-40-Fe and GM-65-Fe exhibit FI-IR spectra similar to GM-40 and GM-65. This similarity in spectra indicates that the structure of the galactomannan did not degrade or appreciably change during the synthesis process of the complexes. For peak assignments, the band at 2910 cm^−1^ is attributed to the C-H stretching and asymmetric vibrations of CH_3_ and CH_2_. In the GM-40 and GM-65 FT-IR spectra, the bands at 1637 and 1430 cm^−1^ are attributed to the stretching vibration of C=O and C-O, respectively. The absorption of the stretching vibration of C=O and C-O in GM-40-Fe and GM-65-Fe shifted to a lower wavenumber by blue-shifting, at 1605 and 1376 cm^−1^. This may be attributable to the carboxyl groups and hydroxyl groups of galactomannan being involved in iron chelation during the synthesis process [12,24]. Chi et al. [12] speculated that this change might be caused by the formation of polysaccharide–iron(III) complexes. According to the reported work [7,25], the absorption peaks at 840–850 cm^−1^ and 680–690 cm^−1^ were attributed to the β-FeOOH structure in the polysaccharide–iron(III) complexes. The bands at 850 and 680 cm^−1^ in Figure 3 were found in the spectra for GM-40-Fe and GM-65-Fe, while they cannot be seen in the spectra of GM-40 and GM-65. These findings indicate that the polymerized β-FeOOH iron cores were present in the galactomannan–iron(III) complexes of GM-40-Fe and GM-65-Fe. Using this information, a schematic diagram of the core–shell structure of galactomannan–iron(III) complexes was speculated and is shown in Figure 1.

**Figure 4:**
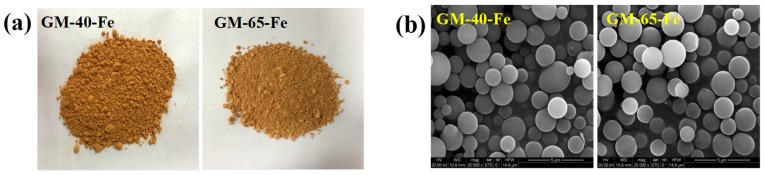

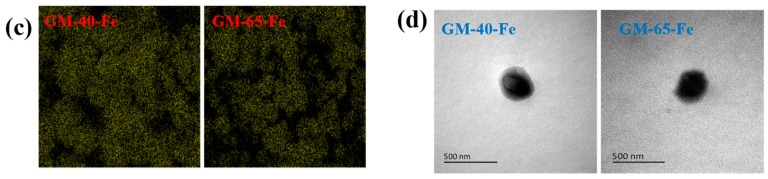
Morphological properties of galactomannan–iron(III) complexes. (**a**): visual images of galactomannan–iron(III) complex; (**b**): SEM images of galactomannan–iron(III) complex; (**c**): the distribution of iron in galactomannan–iron(III) complex; (**d**): TEM images of galactomannan–iron(III) complex).

Figure 4a visually displays that both galactomannan–iron(III) complex preparations were of similarly brown color. The SEM images in Figure 4b revealed that both GM-40-Fe and GM-65-Fe were regularly spherical and both had diameters of 0.5–2 μm. In addition, the distribution of iron in GM-40-Fe and GM-65-Fe was visualized and is shown in Figure 4c. The results revealed that atomic iron was homogeneously distributed in the GM-40-Fe and GM-65-Fe particles, indicating the successful introduction and complexation of iron(III) during the synthesis process. Finally, TEM images (Figure 4d) revealed that both GM-40-Fe and GM-65-Fe were regular spheres with a fuscous nucleus and a tinted shell, providing more evidence of our hypothesized core–shell structure. These observations are in accordance with the results of the FT-IR spectra, which suggested that the β-FeOOH iron cores were indeed present in the galactomannan–iron(III) complexes synthesized according to the applied protocol.

**Figure 5:**
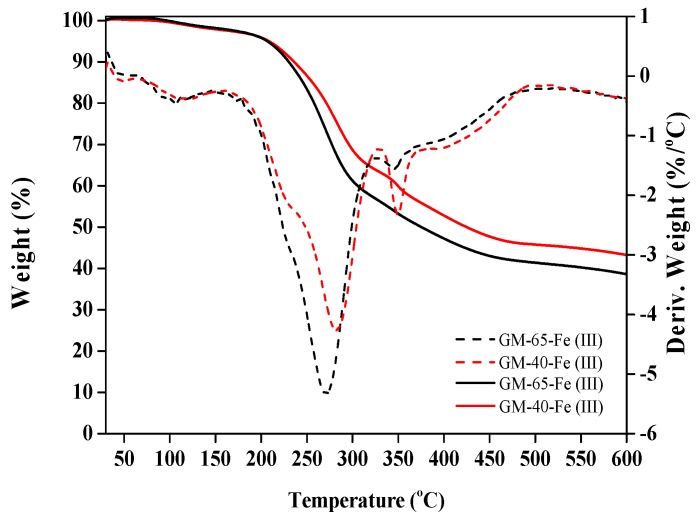
Thermogravimetric (TG) curves and derivative weight (DTG) curves of the galactomannan–iron(III) complexes.

To characterize the thermal behavior of our synthesized galactomannan–iron(III) complexes, thermogravimetric analysis was performed across the temperature range of 30 to 600 °C. The TGA results are shown in Figure 5.

As seen from the thermogravimetric (TG) curves in Figure 5, the thermal decomposition rate of GM-65-Fe was higher than that of the GM-40-Fe at the beginning of the decomposition. This may be related to the greater molecular weight of GM-40-Fe compared to GM-65-Fe. After decomposition, the derivative weight (DTG) curve (derived from the TG curves) showed that the GM-65-Fe and GM-40-Fe demonstrated the maximum decomposition temperature (T_M_) at 270 °C and 285 °C, respectively. In the work of Wen et al. [26], it was also found that the greater molecular weights can lead to T_M_ appearing at a higher temperature. Beyond T_M_, a weak peak at 346 °C was found in the DTG curves of GM-65-Fe and GM-40-Fe. This behavior could be caused by the loss of crystalline water or the phase transition of the iron core in the galactomannan–iron(III) complexes. A similar observation was also noted in the work of Wang et al., [27], who studied the thermal properties of the polysaccharide–iron(III) complexes using polysaccharides from *Angelica sinensis*. It was found that the charred solid residues remaining at 600 °C were 43% for GM-40-Fe and 37% for GM-65-Fe. The difference between the two samples may be explained as the quantity of char residue being inversely proportional to both the iron content and molecular weight of complexes.

### 3.4. Antioxidant Activity of Galactomannan–Iron(III) Complexes

**Figure 6:**
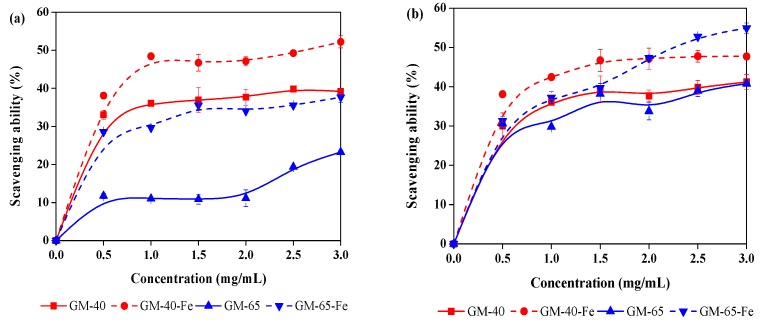
2,2-diphenyl-1-picryl-hydrazyl (DPPH) scavenging ability (**a**) and hydroxyl radical scavenging ability (**b**) of galactomannan and galactomannan–iron(III) complexes.

Galactomannans have been reported as having antioxidant properties that enables scavenging free radicals and reactive oxygen species in vitro. Antioxidant properties are important, as the both free radicals and reactive oxygen species can be responsible for causing many life-threatening diseases [28,29]. Although the galactomannan–iron(III) complexes prepared in this work are intended to be used as organic iron supplements to treat IDA, the galactomannan component in the galactomannan–iron(III) complexes may still demonstrate the aforementioned antioxidant activity. If so, this would lend additional value and an overall uniqueness to the galactomannan–iron(III) complexes that were produced. In order to evaluate whether galactomannan–iron(III) complexes demonstrate the same antioxidant capabilities as galactomannan, both GM-40-Fe and GM-65-Fe were used to scavenge DPPH and hydroxyl radicals. The radical-scavenging assay results are shown in Figure 6. In addition, the antioxidant activity of the original galactomannan preparations (GM-40 and GM-65) were also analyzed for comparison.

As shown in the Figure 6a, both GM-40 and GM-65 can be seen to possess the ability to scavenge DPPH radicals. Unexpectedly, the antioxidant activity of these different preparations of galactomannans was found to be enhanced after being complexed with iron(III) complexes. For example, the DPPH radical scavenging abilities of GM-40 and GM-65 improved from 37.6% to 52.4% and from 23.3% to 39.2% (concentration of 3 mg/mL), respectively. A similar effect was observed regarding the scavenging of the hydroxyl radicals (Figure 6b). For example, 47.7% and 54.9% of the hydroxyl radicals were scavenged by GM-40-Fe and GM-65-Fe, which are higher than that of GM-40 (40.2%) and GM-65 (41.1%). In the work of Lu et al. [5], they also reported that synthesized polysaccharide–iron(III) complexes from *Astragalus membranaceus* showed stronger antioxidant activity than the original polysaccharide. Another interesting observation was that the scavenging abilities of GM-40 and GM-40-Fe were higher than that of GM-65 and GM-65-Fe. This phenomenon is best explained by differences in the molecular weight and function groups between GM-40 and GM-65, both of which have positive effects on antioxidant activity [30,31]. Overall, the relatively higher antioxidant activity of the GM-40-Fe and GM-65-Fe suggests that these galactomannan–iron(III) complexes can serve not only as iron supplements to treat IDA, but also function as antioxidants in vivo.

### 3.5. Bioavailability of Galactomannan–Iron(III) Complexes

An important characteristic of organic iron supplements is their ability to quickly release the iron from the polysaccharide–iron(III) complex once they reach the stomach [2,5]. A supplement’s propensity to release iron can be evaluated by carefully designed biological assays that mimic the environments of internal organs. To verify bioavailability of the prepared galactomannan–iron(III) complexes, iron release assays of GM-40-Fe and GM-65-Fe were performed in artificial gastric juice (pH 2.0) and artificial intestinal juice (pH 8.0), separately. The degree of iron release was used as the index to evaluate the bioavailability of the galactomannan–iron(III) complexes.

**Figure 7:**
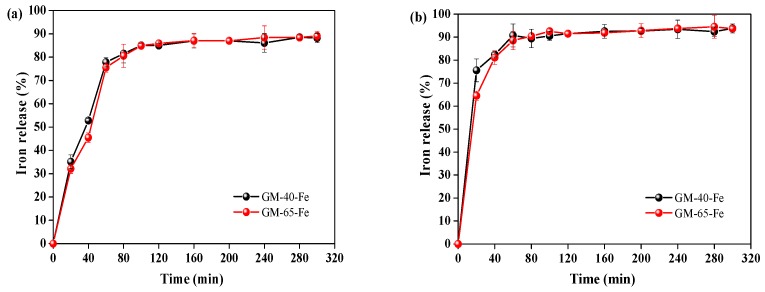
The iron release of galactomannan–iron(III) complexes in the artificial gastric juice at pH 2.0 (**a**) and artificial intestinal juice at pH 8.0 (**b**).

As can be seen in the Figure 7a, 77.8% and 75.5% of iron in GM-40-Fe and GM-65-Fe was released into artificial gastric juice after 60 min, respectively. When the time was prolonged from 60 min to 300 min, further enhancements in the release rates were observed for GM-40-Fe and GM-65-Fe (77.8% to 88.2% and from 75.5% to 88.9%, respectively). When the GM-40-Fe and GM-65-Fe were digested in the artificial intestinal juice (Figure 7b), 89.4% and 90.4% of iron in GM-40-Fe and GM-65-Fe was released after 60 min of incubation, respectively. The rates of iron release from GM-40-Fe and GM-65-Fe were similar to that for the polysaccharide–iron(III) complexes from *Astragalus membranaceus* [5] and *Inonotus obliquus* [2], which were reported as having good bioavailability properties due to their 80–95% iron release rate in similarly conducted assays. Overall, the results in Figure 7 reveal that both GM-40-Fe and GM-65-Fe easily liberate iron during the digestion process in the stomach and the intestines. This indicates that the galactomannan–iron(III) complexes that we prepared have strong propensities to foster iron bioavailability when consumed as organic iron supplements.

## 4. Conclusions

In this work, galactomannan–iron(III) complexes were prepared from different molecular weight galactomannans extracted from *Sesbania* seeds. The characterization results from FT-IR, SEM, and TEM showed that the synthetized galactomannan–iron(III) complexes have β-FeOOH iron cores that are surrounded by galactomannan. Both galactomannan–iron(III) complex preparations demonstrated antioxidant activities as radical scavengers. In all cases, the higher molecular weight complexes were more effective as antioxidants. Finally, the organic iron bioavailability assays revealed that the galactomannan–iron(III) complexes possessed strong bioavailability in simulated gastric and intestinal liquids. This work cumulatively demonstrates that the polysaccharides derived from *Sesbania* seeds can be applied to form organic iron supplements with the added benefit of antioxidant properties.

## Figures and Tables

**Table 1:** Galactomannan content, molecular weight and iron content in galactomannan and galactomannan–iron(III) complexes.

	Mw (g/mol)	Mn (g/mol)	PDI ^1^	Iron Content ^2^ (mg/g)	Galactomannan Content ^2^
GM-40	14,700	12,000	1.23	None	90.9 ± 0.3
GM-65	4890	4380	1.12	None	96.5 ± 0.2
GM-40-Fe(III)	16,100	15,700	1.02	65.4 ± 0.1	74.5 ± 0.1
GM-65-Fe(III)	5320	5270	1.01	57.2 ± 0.1	80.0 ± 0.2

^1^ polydispersity, Mw/Mn; ^2^ Results represents the mean ± standard deviation (SD), *n* = 3.
